# Family Socioeconomic Status and Chinese Adolescents’ Academic Achievement in the Arts: The Mediating Role of Family Arts Resources and the Moderating Role of Family Arts Atmosphere

**DOI:** 10.3389/fpsyg.2021.751135

**Published:** 2021-10-20

**Authors:** Wen Yuan, Hui Guo, C. Victor Fung, Fumei Chen, Lisha Liu, Liangyuan Xu, Yanfang Li

**Affiliations:** ^1^Collaborative Innovation Center of Assessment for Basic Education Quality, Beijing Normal University, Beijing, China; ^2^School of Music, University of South Florida, Tampa, FL, United States; ^3^Center for Teacher Education Research, Faculty of Education, Beijing Normal University, Beijing, China

**Keywords:** family SES, family investment model, gender, urban and rural, academic achievement in the arts

## Abstract

In the present study, we examined the association between family socioeconomic status (SES) and adolescents’ academic achievement in the arts and the mediating and moderating roles of family process factors, verified family investment model. Chinese adolescents (*N* = 8,723) in Grade 8 reported characteristics of family SES, family arts resources, and family arts atmosphere, and then completed a standardized test assessing academic achievement in music and visual art. The results showed that family SES significantly predicted adolescents’ level of academic achievement in the arts after controlling for adolescents’ gender and school location. The effect of family SES on adolescents’ academic achievement in the arts was partly mediated by family arts resources, constituting 20.51% of the total predicted effect. In addition, family arts atmosphere moderated the association between family SES and adolescents’ achievement in the arts. Specifically, family SES had a stronger relationship with academic achievement in the arts for adolescent with higher family arts atmosphere than for adolescent with poor family arts atmosphere. Findings in this study expands the field of influence of the family environments and enhance an understanding of the influence mechanisms of family environments on arts learning.

## Introduction

The arts have long been an important expression of cultural heritage and medium in aesthetic education, which are of great significance in adolescents’ cognitive and emotional development (Hargreaves and Aksentijevic, 2011; Jaschke et al., 2018; Toffalini et al., 2018). UNESCO (2013) took promoting arts education as one of the seven objectives of the LMTF (Learning Metrics Task Force). In China, aesthetic education is also one of the four important educational areas (moral, intellectual, physical, and aesthetic education). According to the *National Curriculum Standards for Arts Education* released by Chinese Ministry of Education (2011), the compulsory arts course in Chinese schools are designated twice or thrice a week. Nevertheless, because of the particular Chinese educational environment (e.g., the pressure surrounding university entrance examinations and limited budgets), in-school arts education in China cannot meet adolescents’ diverse needs for arts learning. To provide a strong complement to school education, families that value arts education highly play a significant role in adolescents’ arts competency training.

In the context of China, many parents emphasize the cultivation of adolescents’ arts competencies and dedicate increasing investments in arts education, such as taking their children to extracurricular arts classes or hiring in-home arts tutors. According to a national survey of 14,206 Chinese parents with nearly 75% have children aged 7 to 14 (Tencent Education, 2012), approximately 63.8% of parents enroll their children in extracurricular arts classes, which is far more than any other type of extracurricular class such as Olympic math, Chinese, and English. Given the substantial parental involvement and investment in children’s arts education, this study explored the extent in which family environments played a role in adolescents’ academic achievement in the arts.

Additionally, students’ academic achievement would be affected by some factors like gender (Wei et al., 2012; Chui and Wong, 2017) and school location (Yusuf and Adigun, 2010; Adesegun et al., 2016). Due to the gamut of diversities found in China, it is necessary to draw a picture of academic achievement in the arts and to consider gender and school location difference in this study.

### Family Socioeconomic Status and Adolescents’ Academic Achievement in the Arts

According to the ecological systems theory (Bronfenbrenner, 1979), family environments, which include family background (e.g., family SES) and processes (e.g., family resources, family atmosphere), play important roles in an individual’s development (Letourneau et al., 2013). Specifically, family SES largely determines basic family characteristics (Williams and Collins, 1995). Many researchers have found that family SES influences children’s development, including physical health (Adler and Ostrove, 1999; Ackerman et al., 2004), cognitive development (Kirsch and Lambert, 2002; Sirin, 2005), and emotional and social adaptation (Guerrero et al., 2006; Hartas, 2011). In particular, SES has been widely found to be closely related to academic performance such as language, reading, math and so on (Kirsch and Lambert, 2002; Lawson and Farah, 2017; Butler and Le, 2018).

On the other hand, according to the Maslow’s hierarchy of needs, only when the basic physiological needs are satisfied will people pursue aesthetic value (Maslow, 1943), which means that high family SES makes it more possible for parents to support their children learning the arts. Some previous studies on family SES and children’s school performance have been focused on music (e.g., Elpus and Abril, 2011; Dell et al., 2015). For example, a study of 1,114 students aged 4–12 in the U.S. who received music training revealed that children from families with low SES had poorer achievement in music than students from families with high SES (Dell et al., 2015). However, we rarely found evidence from mainland China. Considering that children’s arts competency development relies more on family investment than on arts education in schools (Wan, 2009; Tencent Education, 2012; Ministry of Education, 2014), we hypothesize that family SES may have a greater influence on Chinese adolescents’ achievement in the arts.

### Mediating Effect of Family Arts Resources

Socioeconomic status may not only directly affect children’s development but also indirectly affect children’s development through some family processes factors (Coddington et al., 2014). Conger and Donnellan (2007) summarized previous studies and proposed the family investment model that assumed a positive correlation between SES and children’s development, because higher SES allowed parents to provide their children with ample resources. Previous studies of family investment models have mainly focused on children’s physical, cognitive and social development (Linver et al., 2002; Chow et al., 2017; Jiang et al., 2018). In particular, some studies have shown that family resources played a mediating role in the association between SES and children’s academic performance in language (Yeung et al., 2002; Raviv et al., 2004; Lohndorf et al., 2018). For example, Vasilyeva et al. (2018) research with 1,332 students aged 6–8 years in Russia demonstrated that family income influenced children’s word recognition and reading comprehension through family resources.

Nevertheless, there is a lack of research on the mediating impact of family resources on the association between SES and academic achievement in the arts. Only some relevant evidence has suggested that the family music environment, including family material resources (Zdzinski, 2013), affected children’s musical performance and success (Dell et al., 2015). According to the family investment model, we speculate that family SES influences children’s academic achievement in the arts via family arts resources.

### Moderating Effect of Family Arts Atmosphere

In addition to family arts resources, the family arts atmosphere may have great influence on children’s academic achievement in the arts. According to self-determination theorists, a positive family atmosphere may offset or balance disadvantaged factors on adolescents’ developments by giving them more autonomy (Ryan and Deci, 2000). Although there is no authoritative and consistent definition of family arts atmosphere, the definition of family atmosphere is found in the Merriam-Adlerian Psychology (Griffith and Powers, 2007), which states that it is determined by the climate of the relationships that exist among the parents and children, which conveys family value, interest, and activities. In this study, we define family arts atmosphere to include parents’ care and attitude for the arts activities and parents’ arts related activities together with their children.

Studies have shown that family atmosphere moderates the relationship between SES and children’s cognitive, behavioral, and emotional development (Mcpherson, 2009; Liu et al., 2018). Moreover, there are two different mechanisms to explain the moderating effect of family atmosphere. Some researchers found that a better family atmosphere may enhance the correlation between SES and children’s development (Sun et al., 2018), which is an enhancement mechanism, as opposed to a compensation mechanism which means that family atmosphere can act as a buffer for the negative effects of low SES on children’s development (Guerrero et al., 2006).

In the field of children’s learning, some studies have also shown that family atmosphere moderates the association between SES and children’s academic achievement. For example, Guerrero et al. (2006) found that family emotional support was a universal strong protective factor against academic difficulties with lower SES. Given the above findings, does the family arts atmosphere also play a moderating role in the relationship between SES and adolescents’ academic achievement in the arts? How is the effect moderated? Examination of these questions will enrich the literature on the influence of family environments and increase our understanding of the mechanism of how SES influences adolescents’ academic achievement in the arts in different family arts atmospheres.

**FIGURE 1:**
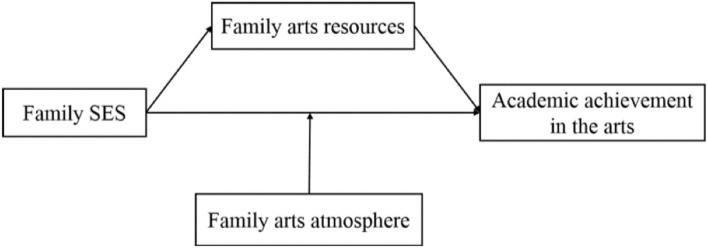
The hypothesized conceptual model.

To summarize, we constructed a model (see Figure 1) that shows the influence of family SES on adolescents’ academic achievement in the arts. Specifically, this study explored the mediating role of family arts resources based on the family investment model and the moderating role of family arts atmosphere. In this study, we sought empirical support and theoretical guidance for the cultivation of adolescents’ arts competence.

## Materials and Methods

### Participants

The participants were selected from 7 provinces in China (Beijing, Tianjin, Jiangsu, Shanxi, Henan, Sichuan, and Gansu). In each province, we adopted a three-stage stratification cluster sampling design. In the first stage, counties were stratified into three layers (upper, middle, and lower) by cluster analysis based on their GDP, urbanization level, and education index. Then, at least 2 counties were extracted from each layer. According to the geographical location and the scale of the school, Probability Proportionate to Size Sampling was used to select schools in each sampling county in the second stage. Then, approximately 10 middle schools were selected from each county. Within each school, approximately 18–20 students in Grade 8 (about 13 years old) were randomly selected. The final sample contained 8,723 adolescents, including 4,681 boys (53.7%) and 4,785 urban adolescents (54.9%).

### Measures

#### Family Socioeconomic Status

Three variables (parents’ educational levels, parents’ occupations, and family possessions) were used to develop family SES (Bradley and Corwyn, 2002). Adolescents reported their parents’ occupations encoded by a four-digit ISCO (International Standard Classification of Occupations) and educational levels (“1 = primary school and below,” “2 = middle school,” “3 = high school,” “4 = college degree,” “5 = bachelor’s degree,” “6 = graduate degree”). We took the average index as the occupation and the higher index as the educational level (Ganzeboom and Treiman, 1996; Ren, 2010). Family possessions were measured by five items adapted from the Program for International Students Assessment (PISA, 2009). Adolescents were asked the following: “Does your family have the following items: (1) TV, (2) washing machine, (3) refrigerator, (4) computer, and (5) personal cars, vans or trucks.” The answer for each item was “1 = zero,” “2 = one,” “3 = two” or “4 = three and above.”

We used exploratory factor analysis (EFA) to create a composite SES (Callan et al., 2017). Only one factor with an eigenvalue greater than one was extracted, accounting for 66.33% of the variance. The factor loading of the parent’s educational level, occupation, and family possession were 0.86, 0.86, and 0.73, respectively. These would suggest that it was reasonable to construct an index of SES (Fabrigar et al., 1999). The mean synthetic index score was 0.16 ± 1.05 (ranging from −2.18 to 3.41). The overall score evaluates students’ SES in the population. This result (*M* = 0.16) indicated that the SES of the sample in this study was higher than that of the population as a whole.

### Family Arts Resources

Family arts resources were measured by five items adapted from the Program for International Students Assessment (PISA, 2009), which assesses the quantity of arts resources in one’s home. Adolescents were asked the following questions: “Does your family have the following items? How many? (1) musical instruments, (2) calligraphy, (3) paintings, (4) arts, and crafts, (5) arts books and picture albums.” The five items were scored as “1 = zero,” “2 = one,” “3 = two” or “4 = three and above.” We computed the total score of all the items (*M* = 10.40, *SD* = 4.25). The higher the scores, the richer the family arts resources. The Cronbach’s α coefficient of the total scale was 0.81.

In addition, we used exploratory factor analysis (EFA) to verify the construct validity of the scale (KMO = 0.81, χ^2^ = 11317.30, *df* = 10, *p* < 0.001). Only one factor with an eigenvalue greater than one was extracted, accounting for 53.85% of the variance. The factor loading of the five items were ranged from 0.64 to 0.76. This would suggest that the scale measures a single construct of family arts resources (Fabrigar et al., 1999).

### Family Arts Atmosphere

The adolescents reported the family arts atmosphere, which was measured by five items adapted from the National Assessment of Educational Progress (National Assessment of Educational Progress [NAEP], 2008). The five items were as followed: “(1) Do your family ask about your arts lessons at school? (2) How often do your parents communicate with you about the arts? (3) Do your family listen to music, sing or play with you? (4) Do your family attend art exhibitions or performances with you? (5) Do your family have a positive attitude toward your participations in art-related activities?” Each item was scored as “1 = never,” “2 = sometimes,” “3 = often” or “4 = always,” and we computed the total score of all the items (*M* = 10.22, *SD* = 3.17). The higher the score, the higher the family arts atmosphere. The Cronbach’s α coefficient of the total scale was 0.78.

In addition, we used EFA to verify the construct validity of the scale (KMO = 0.83, χ^2^ = 14149.48, *df* = 10, *p* < 0.001). Only one factor with an eigenvalue greater than one was extracted, accounting for 57.55% of the variance. The factor loading of the five items were ranged from 0.61 to 0.85. This would suggest that the scale measures a single construct of family arts atmosphere (Fabrigar et al., 1999).

### Academic Achievement in the Arts

According to *National Curriculum Standards for Arts Education* released by Chinese Ministry of Education (2011), the arts compulsory course offered by Chinese schools were mainly music and visual arts. Our study focuses on students’ academic achievement in the arts, so we used these two subjects–music and visual art as the measurement of academic achievement in the arts. The students’ arts achievement was assessed by a standardized test that was developed by a team recruited by National Assessment Center of Education Quality, Ministry of Education, China, including arts education and educational statistics and measurement experts, and outstanding middle school music and visual arts teachers. The arts test measures students’ knowledge and skills in music and visual arts by asking them to observe, describe, analyze, and evaluate works of music and visual arts and to create some original works. The music test included three dimensions: Listening & Identification, Appreciation, and Performance & Creation and the Cronbach’s α coefficient of the music test was 0.74; the visual arts test also included three dimensions: Observation & Identification, Appreciation, and Creation and the Cronbach’s α coefficient of the visual arts test was 0.81. More details about the test can be seen in the Supplementary Files.

Referring to international large-scale evaluation programs (e.g., National Assessment of Educational Progress [NAEP], 2008; OECD, 2010), to estimate students’ academic achievement in the arts more accurately, the Scale Score was used to evaluate academic achievements. This score is based on the IRT (Item Response Theory), which converts the original test score into the ability score, and then converts the students’ ability score into a standard score (*M* = 500, *SD* = 100).

### Procedure

Data on the 8th grade adolescents’ SES, family arts environment and academic achievement in the arts were collected by trained research assistants majoring in psychology or education. All adolescents were measured anonymously in their classrooms after their parents signed the informed consent forms. The recruitment and data collection procedures were approved by the affiliating university’s institutional review board.

## Analysis and Results

We used SPSS to conduct the preliminary analyses and PROCESS macro to explore the hypothetical relationships among family SES, family arts resources and arts atmosphere, and adolescents’ academic achievement in the arts.

First, we computed the descriptive statistics and the Pearson Product-Moment correlation for all variables in this study. Then a 2 × 2 MANOVA was used to examine the effects of gender and school location (urban and rural). We also followed procedures recommended by Hayes (2012), using the PROCESS macro for SPSS, to explore whether the effect of family SES on adolescents’ academic achievement in the arts was mediated by family arts resources and moderated by the family arts atmosphere. The SPSS macro also allowed us to control for the demographic variables (i.e., gender and school location). All effects were tested using the 95% biased-corrected confidence intervals (CI) method, and the number of bootstrapping samples was set to 1,000. Significant indirect effects occur when the 95% CI does not include zero (Hayes, 2013).

### Preliminary Analysis

Table 1 showed means and standard deviation of the variables included in the study. A 2 (gender) × 2 (school location: urban and rural) MANOVA was conducted to examine the differences in family arts resources, family arts atmosphere, and academic achievement in the arts as a function of their gender and school location. Results indicated that girls had significantly higher family arts resources, family arts atmosphere, and academic achievement in the arts than boys (*F* ranged from 117.03 to 195.52, *df* = [1, 8,719], *p* < 0.001). Urban school students’ values for family arts resources and academic achievement in the arts were significantly higher than those of rural school adolescents (*F* ranged from 35.76 to 97.24, *df* = [1, 8,719], *p* < 0.001), while there was no significance between urban and rural school adolescents’ family arts atmosphere. The interaction effect of “gender × school location” on family arts resources, family arts atmosphere, and academic achievement in the arts was non-significant.

**TABLE 1:** Descriptive statistics (means and standard deviations) among gender and school location.

		**Family arts resources**	**Family arts atmosphere**	**Academic achievement in the arts**
Gender	Boy	9.94(4.20)	9.84(3.09)	509.90(92.14)
	Girl	10.94(4.25)	10.67(3.21)	538.18(96.19)
School location	Urban	11.12(4.33)	10.44(3.22)	542.39(93.53)
	Rural	9.54(3.98)	9.96(3.09)	499.45(91.59)
Total		10.40(4.25)	10.22(3.16)	523.00(95.08)

Table 2 showed the correlation matrix of all variables involved. Family SES was positively correlated with family arts resources (*r* = 0.40, *p* < 0.001), family arts atmosphere (*r* = 0.19, *p* < 0.001), and adolescents’ academic achievement in the arts (*r* = 0.19, *p* < 0.001). Family arts resources had strong positive correlations with family arts atmosphere (*r* = 0.54, *p* < 0.001) and adolescents’ academic achievement in the arts (*r* = 0.35, *p* < 0.001). Family arts atmosphere was significantly correlated with adolescents’ academic achievement in the arts (*r* = 0.25, *p* < 0.001). In addition, gender was positively correlated with family arts resources (*r* = 0.12, *p* < 0.001), family arts atmosphere (*r* = 0.13, *p* < 0.001), and adolescents’ academic achievement in the arts (*r* = 0.13, *p* < 0.001); school location was positively correlated with family SES (*r* = 0.43, *p* < 0.001), family arts resources (*r* = 0.19, *p* < 0.001), family arts atmosphere (*r* = 0.08, *p* < 0.001), and adolescents’ academic achievement in the arts (*r* = 0.23, *p* < 0.001). Therefore, gender and school location were taken as control variables in the subsequent analysis to examine the role of family environments more accurately.

**TABLE 2:** Correlation matrix of all study variables.

	**1**	**2**	**3**	**4**	**5**	**6**
1 Gender	–					
2 School location	–	–				
3 Family SES	0.01	0.43***	–			
4 Family arts resources	0.12***	0.19***	0.40***	–		
5 Family arts atmosphere	0.13***	0.08***	0.19***	0.54***	–	
6 Academic achievement in the arts	0.15***	0.23***	0.42***	0.35***	0.25***	–

*Gender (1 = girl, 0 = boy), School location (1 = urban, 0 = rural); ^∗∗∗^*p* < 0.001.*

### Mediating Effect of Family Arts Resources on the Association Between Socioeconomic Status and Adolescents’ Academic Achievement in the Arts

**FIGURE 2:**
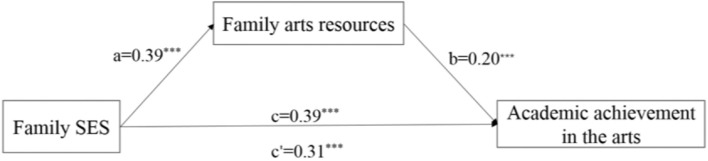
Mediation model of family arts resources. Notes: Control variables were gender and school location. Estimates are standardized coefficients, *p < 0.05, **p < 0.01, and ***p < 0.001.

After controlling for gender and school location, family SES significantly predicted adolescents’ academic achievement in the arts (β = 0.39, *p* < 0.001). The PROCESS macro (Model 4) was used to test the mediating role of family arts resources between family SES and adolescents’ academic achievement in the arts. As shown in Figure 2, family SES significantly predicted family arts resources (β = 0.39, *p* < 0.001, *R*^2^ = 0.17, *F* [3, 8,719] = 610.85, *p* < 0.001). Both family SES (β = 0.31, *p* < 0.001) and family arts resources (β = 0.20, *p* < 0.001) significantly predicted adolescents’ academic achievement in the arts (*R*^2^ = 0.23, *F* [4, 8,718] = 648.86, *p* < 0.001). Further mediation effect tests showed that the association between family SES and adolescents’ academic achievement in the arts was partly explained by family arts resources (β = 0.08, *p* < 0.001, 95% CI = [0.067, 0.085]). The direct effect accounted for 79.7% of the total effect size and the indirect effect of family arts resources accounted for 21.3% of the total effect size, which indicated that 21.3% of the effect of SES on adolescents’ academic achievement in the arts was through family arts resources.

### Moderating Effect of Family Arts Atmosphere on the Association Between Socioeconomic Status and Adolescents’ Academic Achievement in the Arts

**FIGURE 3:**
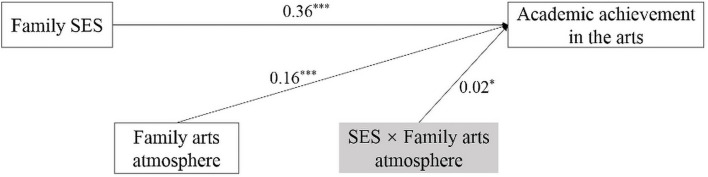
Moderated model of family arts atmosphere. Notes: Control variables were gender and school location. All continuous variables were standardized before they were entered in the path analysis model. Estimates are standardized coefficients, *p < 0.05, **p < 0.01, and ***p < 0.001.

The PROCESS macro (Model 1) was used to test the moderating role of the family arts atmosphere (see Figure 3) on the association between family SES and academic achievement in the arts. After controlling for gender and school location, the interaction term “SES × family arts atmosphere” significantly predicted adolescents’ academic achievement in the arts (β = 0.02, *p* < 0.05, *R*^2^ = 0.22, *F* [5, 8,717] = 495.12, *p* < 0.001, 95% CI = [0.002, 0.039]), which meant that family arts atmosphere played a moderating role in the association between SES and adolescents’ academic achievement in the arts.

**FIGURE 4:**
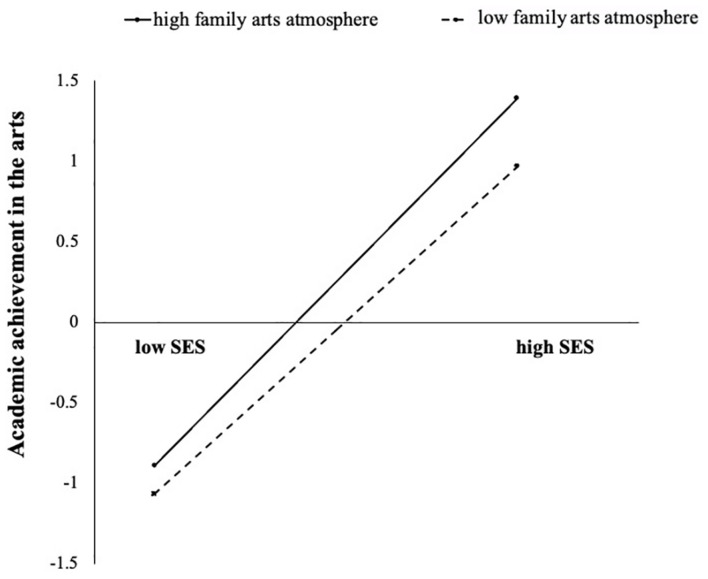
Interaction between family SES and family arts atmosphere in predicting academic achievement in the arts.

To reveal the nature of the moderating role of family arts atmosphere more clearly, we drew a simple effect analysis diagram (Dearing and Hamilton, 2006). The results showed that when the family arts atmosphere was high, family SES had a stronger positive effect on adolescents’ academic achievement in the arts (B_*simple*_ = 0.38, *p* < 0.001, 95% CI = [0.352, 0.410]); when the family arts atmosphere was low, the promotion effect of family SES on adolescents’ academic achievement in the arts was weaker (B_*simple*_ = 0.34, *p* < 0.001, 95% CI = [0.310, 0.368]). In other words, with the improvement in the family arts atmosphere, the effect size of family SES on adolescents’ academic achievement in the arts increased. Therefore, the interaction pattern conformed to enhancement mechanism (see Figure 4).

## Discussion

This study investigated the role of family SES on adolescents’ academic achievement in the arts and the related mechanisms. The results showed that family SES significantly predicted adolescents’ academic achievement in the arts, and the direct effect was mediated by family arts resources and moderated by family arts atmosphere, which enriched the field of influence of the family environment and enhanced the understanding of arts learning mechanisms.

### Main Results Discussions

The current study found gender differences and school location differences in adolescents’ academic achievement in the arts. First, female adolescents’ academic achievement in the arts was significantly higher than the male counterpart, which was consistent with the results of previous studies on gender differences in academic performance (Cenk and Demir, 2016; Alghadir et al., 2020). In the process of gender socialization, parents played an important role and showed different educational expectation and behaviors on boys and girls (Chui and Wong, 2017), which may led to girls prefer the arts and boys prefer sports. Specifically, we found that girls had more family arts resource and higher family arts atmosphere than boys in our study. All of these contributed to gender difference in academic achievement in the arts. Meanwhile, adolescents in urban school locations have higher academic achievement in the arts than adolescents in rural school locations, which was consistent with the results of previous studies on urban-rural differences in academic performance (Yusuf and Adigun, 2010; Adesegun et al., 2016). A possible reason for this situation is that, compared with schools in rural areas where the economy and culture are relatively less modernized (China Statistical Yearbooks, 2018), Chinese urban schools can utilize more arts resources, such as arts galleries, theaters, and museums.

Consistent with our hypotheses and prior research conducted in other academic subjects, such as language, reading, and math (Kirsch and Lambert, 2002; Raviv et al., 2004; Letourneau et al., 2013; Butler and Le, 2018), the current study indicated that SES influenced adolescents’ academic achievement in the arts both directly and indirectly through family arts resources after controlling for adolescents’ gender and school locations. In agreement with the family investment model (Conger and Donnellan, 2007), our findings underline the crucial role of family investments in predicting individual differences in adolescents’ academic achievement in the arts. These results obtained by testing the mediation model in a Chinese sample suggested that the family investment model could be applied universally (Adler and Ostrove, 1999; Linver et al., 2002; Vasilyeva et al., 2018). Based on Maslow’s hierarchy of needs (Maslow, 1943), only parents in families with higher SES can afford to invest money and time in their adolescents’ arts learning, so their adolescent have more opportunities for arts edification and training. As China is a developing country, Chinese families play an important role in adolescents’ arts learning because of the limited arts training in the schools. Meanwhile, the social reality of the widening SES distribution gap of Chinese residents means that families in some areas are still struggling with poverty (China Statistical Yearbooks, 2018). For families with low SES, surviving or pursuing better living conditions is the top priority, and parents do not pay much attention to their adolescents’ arts learning.

Another novel finding of the current study was that the family arts atmosphere moderated the direct effect of family SES on adolescents’ academic achievement in the arts, which was consistent with previous studies in other fields (Coatsworth and Masten, 1998; Taylor and Seeman, 1999; Bradley and Corwyn, 2002). As the family arts atmosphere increased, the predicted effect of family SES on adolescents’ academic achievement in the arts also increased, which can be attributed to an enhancement effect. Research has shown that adolescents were more likely to develop strong intrinsic musical motivations when they lived in warm, caring and non-threatening home environments (Mcpherson, 2009). Adolescents with a better family arts atmosphere receive more encouragement to participate in the arts activities from their parents and have more intrinsic motivations to learn arts (Ryan and Deci, 2000), which ultimately contributes to the promotion of family SES on adolescents’ academic achievement in the arts. As shown in the survey above, many Chinese parents send their children to extracurricular arts classes (Tencent Education, 2012), but they think that it is enough to have professional teachers to guide their children and do not pay attention to the creation of family arts atmosphere (Chao Heng Arts School, 2017). In this context, it is of substantial practical importance to promote Chinese parents’ awareness of creating a good family arts atmosphere, which will enhance the effect of family SES in adolescents’ arts achievement.

### Application, Limitations and Directions for Future Research

These results point to a specific direction for parents whose target is to improve their children’s arts learning. Parents can provide richer family arts resources, such as musical instruments and art works, to enhance children’s arts competencies. In addition, all parents should pay attention to the cultivation of family arts atmosphere, especially those in low SES, who can create a supportive family arts atmosphere (e.g., talking about arts with their children, singing or drawing with their children) to buffer the less than ideal impact of family background on children’s arts learning. For arts teachers, they can guide parents to attach great importance on family arts education by means of educating parents through school publicity, to create and encourage a good family arts atmosphere for their children.

Based on the current findings, we recommend a few directions for further investigations. First, since data in this study were cross-sectional, it was not possible to draw clear conclusions about the causal relationship among the variables. To further determine the models of family environment and adolescents’ arts development, it is necessary to carry out longitudinal designs or experimental studies. Second, the use of self-reported data in this study might have given rise to false reporting because of social expectations. Future studies should consider collecting reports from parents and teachers. Thirdly, ecological systems theory points to multiple sociocultural processes outside family environments, such as schools and communities. Future researchers can investigate these factors, which may complement the mechanisms on the associations of family environments and adolescents’ arts performance.

## Data Availability Statement

The raw data supporting the conclusions of this article will be made available by the authors, without undue reservation.

## Ethics Statement

The studies involving human participants were reviewed and approved by the Collaborative Innovation Center of Assessment toward Basic Education Quality, Beijing Normal University. Written informed consent to participate in this study was provided by the participants’ legal guardian/next of kin.

## Author Contributions

WY designed the study, analyzed the data, and wrote the manuscript. HG collaborated in designing the research. CVF collaborated in manuscript revisions. FC collaborated in manuscript revisions and research theme design. LL assisted with designing the research and manuscript revisions. LX assisted with designing the research. YL provided overall guidance. All authors contributed to the article and approved the submitted version.

## Conflict of Interest

The authors declare that the research was conducted in the absence of any commercial or financial relationships that could be construed as a potential conflict of interest.

## Publisher’s Note

All claims expressed in this article are solely those of the authors and do not necessarily represent those of their affiliated organizations, or those of the publisher, the editors and the reviewers. Any product that may be evaluated in this article, or claim that may be made by its manufacturer, is not guaranteed or endorsed by the publisher.
