# Supplementary material for: Family Socioeconomic Status and Chinese Adolescents’ Academic Achievement in the Arts: The Mediating Role of Family Arts Resources and the Moderating Role of Family Arts Atmosphere
Source: Front Psychol. 2021 Oct 20;12:751135. doi: 10.3389/fpsyg.2021.751135 (PMC8565372; doi:10.3389/fpsyg.2021.751135)
Supplement: Supplementary file 1 [file Data_Sheet_1.pdf]

Supplementary materials

The supplementary materials include two aspects. One is the supplementary information presentation table of the results in the paper, the other is the introduction and sample items of students' arts academic achievement standardized test.

I The tables

Table 1. MANOVA results in respect of school location of and gender (N=8723)

Source	Family arts resources			Family arts atmosphere			Academic achievement in the arts		
	MS	df	*F*	MS	df	*F*	MS	df	*F*
School location	563.50	1	35.76***	2.77	1	.29	749056.76	1	97.24***
Gender	1844.24	1	35.76***	1274.90	1	97.24***	1513891.89	1	6.56*
Gender× School location	23.08	1	1.47	20.13	1	2.10	14672.17	1	1.91
Error	15.76			9.60			7703.47		

Table 2 Results of the mediation analysis with family arts resources as the mediator

	family arts resource			Academic achievement in the arts		
	β	*p*	95%CI	β	*p*	95%CI
Gender	-.24	.000	[-.279, -.203]	-.26	.000	[-.296, -.221]
School location	.03	.150	[-.011, .074]	.11	.000	[.067, .149]
Family SES	.39	.000	[.368, .410]	.31	.000	[.292, .336]
Family arts resources				.20	.000	[.176, .217]
R^2	.17			.23		
df	[3, 8719]			[4, 8718]		
F	610.85			648.86		
p	.000			.000		

Table 3 Path coefficients representing mediation of family arts resources

Paths in the model	Effect	*p*	95% CI
SES →Academic achievement in the arts (direct effect)	0.39	0.000	[.292, .336]
SES →family arts resources →Academic achievement in the arts	0.08	0.000	[.067, .085]

Table 4 Results of the moderation analysis with family art atmosphere as the moderator

	β	*p*	95%CI
Gender	-.27	.000	[-.303, -.228]
School location	.12	.000	[.076, .159]
Family SES	.36	.000	[.338, .380]
Family arts atmosphere	.15	.000	[.134, .172]
Family SES ×Family arts atmosphere	.02	.034	[.002, .039]
R^2		.22	
df		[5, 8717]	
F		495.12	
p		.000	

Table 5 The effects of moderation analysis with different level of family arts atmosphere

Family arts atmosphere	Effect	*p*	95%CI
M-1SD	.34	.000	[.310, .368]
M	.36	.000	[.338, .380]
M+1SD	.38	.000	[.352, .406]

II The arts academic achievement standardized test

The arts test has taken one and a half years to develop. During the development of the test, the experts took the *National Curriculum Standards for Arts Education* (2011) in the compulsory stage (i.e., grades 1-9) as the reference and selected common knowledge and skills from the textbooks that the selected 7 provinces used in the school as the test content. After multiple modifications, a preliminary test for academic achievement in the arts was formed. Then, two rounds of pilot studies were carried out. According to the difficulty and differentiation measures of each item, we modified and screened the test. Finally, the standardized test assessing academic achievement in the arts was formed.

Before the formal test, provincial supervisors would be sent to the sample school to ensure the test preparation such as test conditions, testers, and so on. Besides, to ensure the authenticity of data, the testers were not employed by the testing school. Half an hour before the test, the testers unseal the list and check the students on site to ensure a random selection of the sample. Arts education experts, teaching and research staff, the forefront of arts teachers and doctoral students in arts completed the scoring process together.

Take the music test (included 44 items) for example, the following provides sample items on three dimensions of the music test.

Listening sample:

Listen to the song and answer the following two questions (Play only once):

(1) Which region of our country does the song belong to?

A. Northeast B. Jiangsu and Zhejiang provinces C. Northwest D. Southwest

Correct answer: C

(2) What's the genre of this song?

A. Folk song B. Horn C. Popular tune D. Toasting song

Correct answer: A

Audio's name: *Driving Livestock*

Appreciation sample:

Listen to the song "*Defend the Yellow River*". You should use your musical knowledge to describe the techniques used in this song that express the strong determination and triumphalism of the Chinese people resisting aggression. Please fill out the answer sheet. (Play it twice)

**Score**	**Criteria**
3	Wrote out sing form(chorus or troll) and 2 other points (like timbre,instruments,lyrics,emotion,rhythm,meter, dynamics tempo, genre, melodic progression, tonality etc.).<br>**Or** not wrote out sing form(chorus or troll) but 4 other points (like timbre,instruments,lyrics,emotion,rhythm,meter,dynamics tempo, genre, melodic progression, tonality etc.)
2	Wrote out sing form(chorus or troll) and 1 other points (like timbre,instruments,lyrics,emotion,rhythm,meter, dynamics tempo, genre, melodic progression, tonality etc.).<br>**Or** not wrote out sing form(chorus or troll) but 2-3 other points (like timbre,instruments,lyrics,emotion,rhythm,meter,dynamics tempo, genre, melodic progression, tonality etc.) .<br>**Or** only wrote out sing form(chorus or troll) .
1	Wrote out sing form(chorus or troll) but 1 other points (like timbre,instruments,lyrics,emotion,rhythm,meter,dynamics tempo, genre, melodic progression, tonality etc.) .
0	No answer; wrong expression

Creation sample:

Please complete the last four bars of the following melody, so that the beat is accurate, the melody is smooth and has a sense of closure. Please note that either the staff or the notation is compiled and the answers are filled in on the answer card.

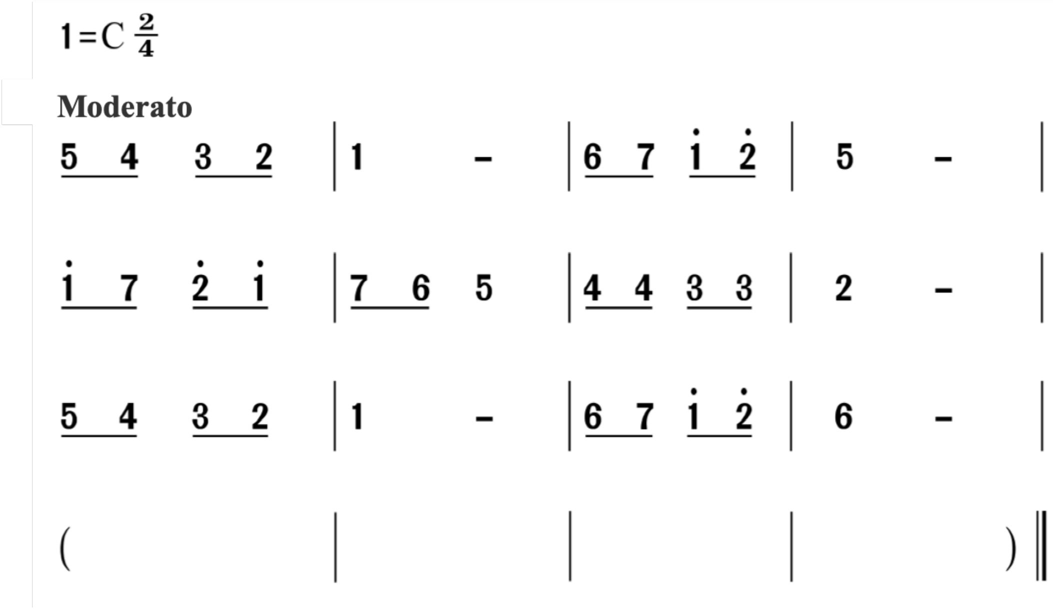

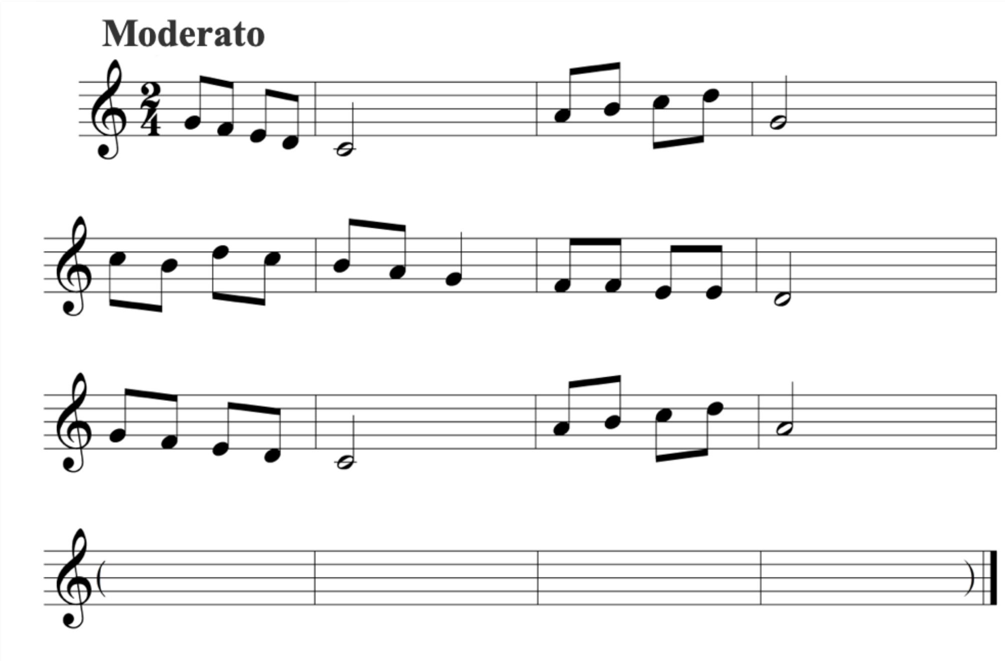

Score	Criteria
3	The rhythm and meter were accurate, and the notes were written with basic standard in 4 bars; the melody sound smooth and had cadence.
2	The rhythm and meter were accurate, and the notes were written with basic standard in 3-4 bars; the melody sound not smooth but had cadence.<br>**Or** the rhythm and meter were accurate, and the notes were written with basic standard in 3-4 bars; the melody sound smooth but not had cadence.
1	The rhythm and meter were accurate, and the notes were written with basic standard in 3-4 bars; the melody sound not smooth and not had cadence.<br>**Or** the rhythm and meter were accurate, and the notes were written with basic standard in 2 bars; the melody sound not smooth but had cadence.<br>**Or** the rhythm and meter were accurate, and the notes were written with basic standard in 2 bars; the melody sound smooth but not had cadence.
0	No answer.<br>**Or** Wrote out wrong notion.<br>**Or** copy continuously in 4 bars;<br>**Or** the rhythm and meter were not accurate, and the notes were not written with basic standard in 4 bars.<br>**Or** the rhythm and meter were accurate, and the notes were written with basic standard in 1-2 bars; the melody sound not smooth and not had cadence.
